# Spectrally Encoded Confocal Microscopy for Guiding Lumpectomy

**DOI:** 10.1155/2014/573851

**Published:** 2014-11-19

**Authors:** Elena F. Brachtel, Barbara L. Smith, Guillermo J. Tearney, Dongkyun Kang

**Affiliations:** Massachusetts General Hospital, 55 Fruit Street, Boston, MA 02474, USA

## Background

Complete removal of breast cancer during a single breast-conserving lumpectomy procedure is often challenging due to the lack of adequate intraoperative tools to accurately determine the margin status. About one-third of lumpectomy patients are found to have positive margins upon final histologic analysis, which usually is reported within a week after surgery. These patients are then required to undergo additional surgeries, which increases the patient morbidity, cosmetic challenges, and healthcare cost. Spectrally encoded confocal microscopy (SECM) is a high-speed confocal microscopy technique [1] that can visualize cellular and subcellular features of an unstained fresh tissue. SECM is 10–100 times faster than conventional confocal microscopes and has been demonstrated to image an entire endoscopic mucosal resection (EMR) esophageal tissue (10 mm by 10 mm) within 15 seconds [2]. The high imaging speed of SECM may make it possible to rapidly image the margins of entire lumpectomy specimens to comprehensively determine margin status without sampling error. Real-time feedback regarding the margin status could enable the surgeon to achieve more thorough tumor removal in a single surgery and will significantly reduce the need for additional surgeries. The aim of this preliminary study was to test SECM for visualizing breast cancers with various morphologic features.

## Method

We imaged 44 fresh breast tissue samples with SECM and compared the SECM images with corresponding histologic slides. The specimens were obtained from mastectomy and lumpectomy operations conducted at MGH and according to an IRB-approved protocol. Each specimen was immersed in 5% acetic acid to increase the nuclear contrast in SECM. Large-area SECM images (4 mm by 2 mm to 10 mm by 4.8 mm) were obtained at 5 imaging levels (imaging depth: 0 to 200 *μ*m). After SECM imaging, each specimen was processed and cut as hematoxylin and eosin (H&E) stained slides. SECM images were analyzed in comparison with the corresponding H&E slides to identify features in the SECM images that distinguished benign from malignant breast tissues.

## Results

**Figure 1:**
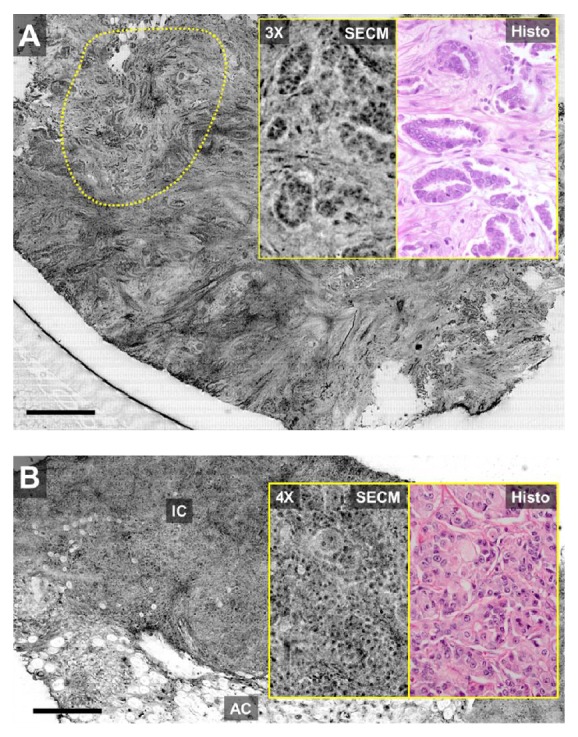
SECM images of low-grade invasive ductal carcinoma (A) and high-grade invasive carcinoma (B). Dotted region in (A) shows small glands invading the stroma in a disorganized fashion. IC—invasive carcinoma; AC—adipose cells. Scale bars = 0.5 mm.

SECM images enabled the visualization of architectural and cellular features of normal and diseased breast tissues that were structurally similar to those seen by histopathology. SECM images of normal breast tissues showed morphologic features of adipose cells and benign glands. Low-grade invasive ductal carcinoma showed small glands invading the stroma in a disorganized fashion (Figure 1(a)). High-grade invasive carcinoma exhibited pleomorphic tumor cells with mostly little stroma (Figure 1(b)).

## Conclusion

Results from this preliminary imaging study showed that SECM can be used to visualize key histomorphologic features associated with breast cancers. We are currently conducting a validation study of testing sensitivity and specificity of SECM for diagnosing breast cancers. When utilized during lumpectomy, SECM may have the potential to rapidly provide margin status and help the surgeon achieve more thorough removal of the tumor in a single surgery.
